# A Randomized, Double‐Blind, Placebo‐Controlled Study to Evaluate the Safety and Efficacy of a Nutraceutical Supplement With Standardized Botanicals in Males With Thinning Hair

**DOI:** 10.1111/jocd.16778

**Published:** 2025-01-06

**Authors:** Neal Bhatia, Glynis Ablon, Patricia K. Farris, Adina Hazan, Isabelle Raymond

**Affiliations:** ^1^ Therapeutics Clinical Research San Diego California USA; ^2^ Ablon Skin Institute Research Center Manhattan Beach California USA; ^3^ Tulane University School of Medicine New Orleans Louisiana USA; ^4^ Nutraceutical Wellness Inc. New York New York USA

**Keywords:** botanicals, hair thinning, male pattern hair loss, MPHL, nutraceutical, supplement

## Abstract

**Background:**

Hair thinning in men is a prevalent issue for which treatment oftentimes consists of a multi‐modal approach. Targeting key root causes of hair thinning, such as hormones, stress, and metabolism through vitamins, minerals, and botanicals, has been shown to be effective in improving hair growth and quality in women. This approach could also be effective in improving hair growth and quality in men with thinning hair.

**Aims:**

This study investigates the safety and efficacy of a nutraceutical in improving hair growth and quality in men with thinning hair.

**Methods:**

This was a 6‐month multi‐center, double‐blind, randomized, placebo‐controlled trial. Men aged 21–61 years old with confirmed hair thinning were included. Subjects were randomized to receive the oral supplement or placebo. The study end points included changes in blinded Global Investigator Ratings for hair growth and quality, the Men's Hair Growth Questionnaire (MHGQ), and changes in the Arizona Sexual Experience Scale (ASEX) in the active group compared to baseline and placebo.

**Results:**

Daily intake of the nutraceutical resulted in significantly more subjects in the active group compared to the placebo rated as improved for hair growth and quality in the blinded investigator global assessments. Overall changes and responses to the MHGQ also corroborated blinded investigator ratings. There were no trends in either treatment group for changes in sexual function on the ASEX questionnaire, and the supplement was well tolerated.

**Conclusions:**

Ingestion of a nutraceutical targeting key root causes of male hair thinning significantly improves hair growth and quality in men with no changes in sexual function.

## 1 Introduction

According to the American Hair Loss Association, hair loss and thinning affect two‐thirds of men by age 35 and can significantly impact an individual's quality of life [1]. While available therapy options have increased over recent years, oftentimes, treatment will involve a multi‐modal approach, including over‐the‐counter options such as topical minoxidil or prescription oral medication like finasteride. Other treatments include low‐level light therapy, platelet‐rich plasma injection, or hair transplants depending on the level of hair loss [2]. And, although genetics have long been known to play a major role in male hair loss and thinning, other factors such as hormones, stress, and environmental influences have now been recognized as key contributing factors [3]. Addressing these contributing factors through botanicals, vitamins, and minerals has been shown to be effective in women, women going through the menopausal transition, and women leading a vegan lifestyle [4, 5, 6, 7, 8]. There are also botanicals with evidence for addressing key root causes of hair thinning in men [5, 9]. Saw palmetto, for example, has been used as an alternative treatment for benign prostatic hyperplasia (BPH) and now has evidence for benefits in hair growth [10, 11]. Its main mechanism of action is thought to be through the inhibition of 5‐a‐reductase, the enzyme responsible for the conversion of testosterone to dihydrotestosterone (DHT), which is thought to play a key role in driving hair thinning [10]. Stress has been linked to hair thinning through cortisol activity at the hair follicle, which is associated with catagen induction and follicular regression [12, 13]. Ashwagandha, an ayurvedic herb traditionally used to reduce stress, has been shown to mitigate systemic cortisol fluctuations [12]. Similarly, there is evidence to suggest that oxidative stress at the scalp and hair follicle leads to damage to the amino acids of the hair [14]. Taking supplements with nutrients that provide antioxidant activity, such as vitamin E and curcumin, has been shown to decrease oxidative stress markers in humans [4, 15]. In this way, using botanicals with evidence of addressing the key underlying drivers of hair thinning should improve hair growth and quality [9, 14]. More so, combining multiple clinically studied ingredients into a single supplement at efficacious levels should be a reasonable approach to improve men's hair growth and quality in men with thinning hair.

Interest in complementary or alternative medicine is on the rise and is increasingly being integrated into conventional medical practices [16]. Male patients in the early stages of thinning make up a large portion of the population exploring supplements to address hair thinning, with 61% of the global hair supplement and treatment market in 2023 driven by men [17]. Moreover, the market share of hair growth supplements and treatments is estimated to grow by a 6.1% compound annual growth rate between 2024 and 2032 [17]. As part of this trend, a rising number of supplements addressing hair thinning are becoming commercially available, yet clinical data on their efficacy and safety remain scarce. And, while the Food and Drug Administration (FDA) regulates the supplement industry for ingredient safety, adverse events (AEs), and claims, supplement companies are not required to conduct or submit to FDA clinical efficacy studies prior to being sold on the market. In this way, men searching for additional or complementary therapies to address hair thinning may be subject to unreliable or unsafe products whose formula is not tested for safety and/or efficacy.

In this study, a standardized nutraceutical targeting key underlying drivers of hair thinning in men identified as hormones, stress, oxidative stress, nutrition, aging, and metabolism was evaluated for safety and efficacy in men with hair thinning. The nutraceutical was composed of saw palmetto, ashwagandha, vitamin E, curcumin, and other botanicals, vitamins, and minerals with evidence to support hair growth in men. And while therapies addressing hair thinning in men are generally given in combination, here we evaluate the supplement alone.

## 2 Materials and Methods

This was a 6‐month multi‐center, randomized, double‐blind, placebo‐controlled study in men with hair thinning (ClinicalTrials.gov identifier: NCT05339958). Healthy men with self‐perceived hair thinning were included in the study. All participants provided written, informed consent before engaging in any study‐related procedures. All investigations were performed in accordance with the rules of the Declaration of Helsinki of 1975, under the approval of an Institutional review board (Advarra, Columbia, MD, USA), and conducted in compliance with good clinical practice.

### 2.1 Subject Selection

Inclusion criteria were males in good health between 21 and 55 years of age with self‐perceived thinning hair. Hair thinning was clinically confirmed by a dermatologist and limited to Levels II, IIA, III, IIIv, and IV using the Norwood–Hamilton (NH) classification of patterned hair loss.

Exclusion criteria were individuals with clinical diagnosis of hair loss disorder such as alopecia areata, or scarring forms of alopecia; subjects with advanced pattern hair loss; scalp hair loss at the treatment area, due to disease, injury, or medical therapy; current skin disease; history of surgical correction of hair loss on the scalp (i.e., hair transplant); use of any products or devices purported to promote scalp hair growth (e.g., finasteride or minoxidil) within the 6 months; use of anti‐androgenic therapies or use of low‐level lasers for hair growth within 3 months; history of malignancy; a known history of autoimmune thyroid disease, any other thyroid disorder/abnormality or other autoimmune disorders; known history or recent blood work indicating iron deficiency, bleeding disorders or platelet dysfunction syndrome; previously experienced serious complications due to COVID‐19 and smokers with usage > 20 cigarettes/day.

### 2.2 Study Procedures

Once subject eligibility was verified, subjects were randomized to either the hair nutraceutical (Nutrafol Men's Capsules, Nutraceutical Wellness Inc., New York, NY, USA) or the placebo in a 2:1 active: placebo ratio. Subjects were instructed to take four capsules once daily with a meal. Both investigational products were identical in appearance and placed in pre‐labeled bottles kept blinded to the investigators and subjects throughout the course of the study. The 6‐month study consisted of three clinic visits at baseline, Day 90 and Day 180, and compliance calls on Days 45 and 135. At each clinic visit, a general physical examination, global photographs, hair pull test, and questionnaires were done. Specifically for the hair exam at Visit 1 (Day 0), the scalp was examined by the investigator to rule out any confounding scalp conditions.

Two‐dimensional standardized global photographs were obtained using IntelliStudio Canfield Scientific Standardized Global Photography. A total of eight standard images were taken of the entire head, hair, and target regions. Blinded global investigator assessments of change in hair growth and quality were evaluated using the standardized global photographs [18]. Ratings were based on a 7‐point Likert scale (−3 greatly worsened to +3 greatly improved) compared to baseline images [18]. Hair quality was defined as hair brittleness, dryness, texture, shine, scalp coverage, and overall appearance.

The hair shed pull test was used to assess active shedding at all clinical visits. The investigator applied gentle traction in a group of approximately 60 hairs from proximal to distal end and counted the number of hairs that came out. This was done in four regions: vertex, occipital, right, and left parietal. If ≥ 6 hairs were released in any one section or ≥ 15 total hairs were recorded as shed when summed across all regions, the subject was considered to have a positive pull test indicating active hair shedding. Subjects were instructed not to wash their hair 24 h prior to the visit.

Multiple subject questionnaires were also utilized in this study. The Men's Hair Growth Questionnaire (MHGQ, license 2202664) along with self‐assessments of hair growth, quality, satisfaction, and potential side benefits were captured on Days 90 and 180 [19]. The Arizona Sexual Experience Scale (ASEX, license ID CBJCHBCAABAA_k5J7HpkuRJrhESK2lX5pQfbELCFEhtr) was administered at all timepoints [20]. Per the ASEX, increasing sexual dysfunction is indicated through higher scores, with an individual total score (sum of responses) of ≥ 19, a score of ≥ 5 on any single item, or a score of ≥ 4 on three items. The Perceived Stress Scale (lic. 2301345) was also administered at all time points to document potentially confounding effects of stress on hair, such as Telogen Effluvium, related to the ongoing COVID‐19 pandemic during the study period [21, 22]. The PSS is a 10‐item questionnaire with each question scored from 0 (*never*) to 5 (*very often*). The total possible score range of 0–40 indicates levels of perceived stress, with 0–13 considered low, 14–26 moderate, and 27–40 high. Finally, questionnaires on quality of life and ease of use were administered at baseline and Day 180. AEs were documented and compiled at all visits and compliance calls beyond baseline.

### 2.3 Study End Points

The original primary end point of this 6‐month study was the change in mean terminal hair counts at Day 180 using the Canfield HairMetrix noninvasive device. However, during clinical site monitoring, numerous protocol deviations specifically related to the Canfield HairMetrix occurred at one site, rendering many phototrichograms unusable for analyses, leading to insufficient data to conduct proper statistical analyses. No other data were affected. Therefore, only secondary end points are presented here and include the change in blinded global investigator ratings for hair growth and quality at Day 180 compared to baseline and placebo. Also, changes in hair shedding and responses to the administered questionnaires, including the MHGQ, the Perceived Stress Scale, the ASEX, and other subject assessments, were compared to baseline and/or placebo.

### 2.4 Statistical Analysis

For continuous outcome measurements across three evaluation points of baseline, Day 90, and Day 180, an analysis of covariance was performed with Tukey HSD analysis within each treatment group. Continuous effects between two groups were evaluated using Student's *t*‐test for two correlated samples within each treatment group. For comparison effects between two groups, a Student's *t*‐test for two independent samples was used. Assessment of proportions or categorical measures was evaluated using Fisher's exact test or chi‐square test, within and between treatment groups.

## 3 Results

### 3.1 Demographics and Baseline Characteristics

A total of 112 subjects were enrolled in the study between July 2021 and October 2022, with 85 (52 active and 33 placebo) completing the study per protocol. Reasons for discontinuation included loss to follow‐up (*N* = 14), subject withdrawal (*N* = 9), protocol deviation (*N* = 1), probably related AE (moderate gastrointestinal upset *N* = 2), and unrelated AE (*N* = 1).

The mean age was comparable for each group with 38.4 ± 11.6 (range 21–61) for the active group, and 42.7 ± 11.0 (range 25–61) for the placebo (*p* > 0.05). The per‐protocol baseline demographics are shown in Table 1. A disparity was noted in that there were six subjects of the American Indian race randomized to the active supplement group but none in the placebo group.

**TABLE 1:** Baseline demographics of per protocol population (*n*/%).

Ethnicity	Nutrafol (*n* = 52)	Placebo (*n* = 33)
Hispanic/Latino	16/31%	8/24%
Not Hispanic/Latino	36/69%	25/76%

Race	Nutrafol (*n* = 52)	Placebo (*n* = 32^a^)
Caucasian	39/75%	28/88%
American Indian	6/12%	—
Asian	5/10%	3/9%
African American	2/3%	1/3%

^a^ One subject did not respond.

NH classifications of the subjects are shown in Table 2. NH classification trended higher in the placebo group, with 40% of subjects classified as IV in the placebo group but 23% in the active group. In contrast, the active group had the most subjects classified as III with 37% of the group population, whereas this was only 18% of the population in the placebo group.

**TABLE 2:** Norwood–Hamilton (NH) classification by treatment group (*n*/%).

NH classification	Nutrafol (*n* = 52)	Placebo (*n* = 33)
II	8/15%	7/21%
III	19/37%	6/18%
III vertex	13/25%	7/21%
IV	12/23%	13/40%

### 3.2 Investigator Global Assessment

**FIGURE 1:**
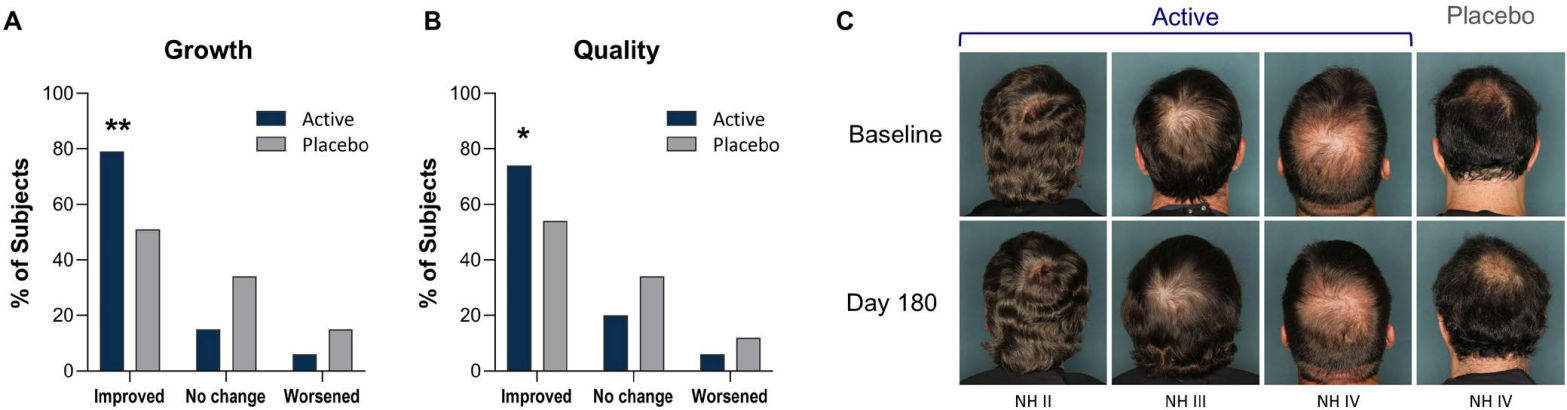
Improvements in hair growth and quality at Day 180 by blinded investigator global assessment. Bar graph showing percent of subjects rated as improved, no change, or worsened in (A) growth and (B) quality. (C) Representative subjects of different Norwood–Hamilton (NH) levels showing improvement in scalp coverage in the active subjects but not in the placebo subjects. *p < 0.05; **p < 0.01 compared to placebo.

Blinded investigator global assessment showed significant improvements in hair growth and quality for the nutraceutical compared to placebo. Indeed, there was a statistically significant improvement in ratings for hair growth compared to placebo at Day 180 (*p* < 0.01, Figure 1). This was a progressive increase from baseline, with 57% of subjects rated as improved at Day 90, increasing to 79% by Day 180 in the active group (*p* < 0.05 Day 180 vs. Day 90). The placebo group had 48% and 51% of subjects rated as improved at Days 90 and 180, respectively, a nonsignificant difference over time (*p* > 0.05).

Likewise, blinded investigator global assessment of quality showed a statistically significant improvement in the active group compared to placebo at Day 180, with 74% rated as improved in the active group compared to 54% in the placebo group (*p* = 0.026). Improvements in the active group were progressive, with 61% rated as improved at Day 90, whereas the placebo group remained consistent at 54% of subjects rated as improved at both time points. In addition, ratings of “no change” or “worsened” trended higher in the placebo group for both growth and quality at both time points compared to the active group, although this did not reach statistical significance (*p* > 0.05, Figure 1).

### 3.3 Hair Pull Test

No subject in either group recorded a positive pull test on the overall scalp, all having < 15 total hairs shed summed across all regions on the head at any time point. A single subject (placebo group) recorded a positive pull (seven hairs) in one region at the baseline time point. These results indicate that the overall subject population in both groups did not have active hair shedding during the study.

### 3.4 Men's Hair Growth Questionnaire

**FIGURE 2:**
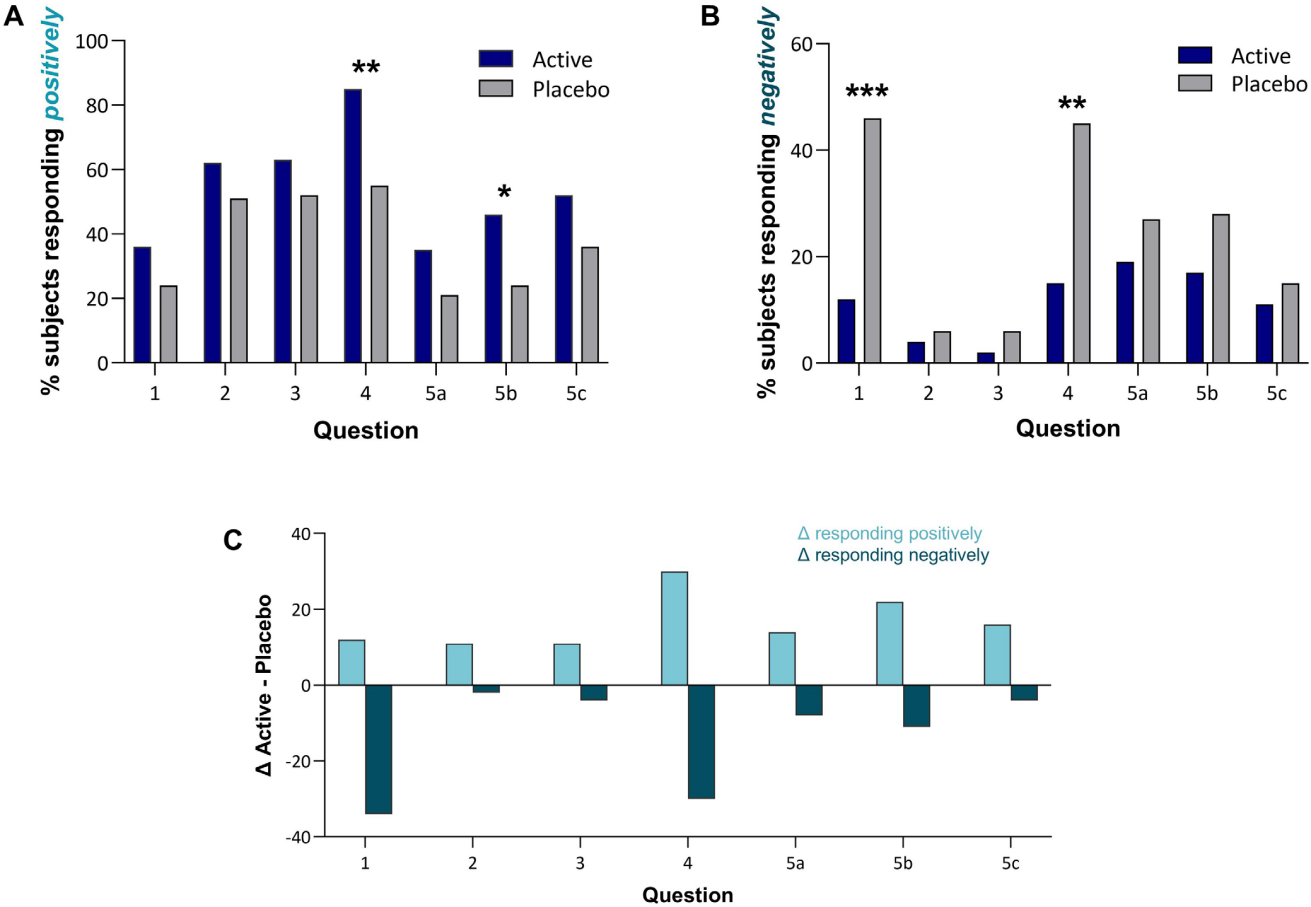
Subject responses to the Men's Hair Growth Questionnaire (MHGQ) at Day 180. (A) Percentage of subjects in active and placebo groups responding *positively* to each question in the MHGQ. (B) Percentage of subjects in active and placebo groups responding *negatively* to each question in the MHGQ. (C) Difference in responses between active and placebo groups in MHGQ. Bars indicating positive responses (light teal) in positive direction indicate that the active group agreed more than the placebo group. Bars indicating negative responses (dark teal) in the negative direction indicate that the placebo group disagreed more than the active group. *p < 0.05; **p < 0.01; ***p < 0.0005 compared to placebo.

The results of the MHGQ show that the men in the active group reported better improvements in hair growth than the placebo group. For all questions, the percentage of subjects responding positively at Day 180 was higher in the active group compared to the placebo group (Figure 2A,C). Likewise, the percentage of subjects responding with a negative rating at Day 180 was lower in the active group compared to the placebo group for all questions (Figure 2B,C). Specifically, the active group had statistically significantly better responses than the placebo group regarding, “how effective do you think the treatment has been in slowing down your hair loss?” (85% vs. 55%, *p* < 0.05) and “satisfied with the appearance of the hair on top of your head” (46% vs. 24%, *p* < 0.05) (Table 3). Conversely, subjects in the placebo group had significantly worse responses than the active group regarding, “I can see my bald spot getting smaller” (46% vs. 12%, *p* < 0.05) (Table 3).

**TABLE 3:** Men's Hair Growth Questionnaire results at Day 180.

	Active	Placebo
1. Since the start of the study, I can see my bald spot getting smaller (agree/disagree).		
% responding positively	36%	24%
% responding negatively	12%***	46%
2. Because of the treatment I have received since the start of the study, the appearance of my hair is (better/worse).		
% responding positively	62%	51%
% responding negatively	4%	6%
3. Since the start of the study, how would you describe the growth of your hair? (increased/decreased)		
% responding positively	63%	52%
% responding negatively	2%	6%
4. Since the start of the study, how effective do you think the treatment has been in slowing down your hair loss? (effective/not effective)		
% responding positively	85%**	55%
% responding negatively	15%**	45%
5a. Compared to the beginning of the study, which statement best describes your satisfaction with the appearance of the hairline at the front of your head? (satisfied/dissatisfied)		
% responding positively	35%	21%
% responding negatively	19%	27%
5b. Compared to the beginning of the study, which statement best describes your satisfaction with the appearance of the hair on top of your head? (satisfied/dissatisfied)		
% responding positively	46%*	24%
% responding negatively	17%	28%
5c. Compared to the beginning of the study, which statement best describes your satisfaction with the appearance of your hair overall? (satisfied/dissatisfied)		
% responding positively	52%	36%
% responding negatively	11%	15%

* *p* < 0.05 compared to placebo.

** *p* < 0.01 compared to placebo.

*** *p* < 0.0005 compared to placebo.

### 3.5 Arizona Sexual Experience Scale

Overall scores for both groups were within normal range at the start of the study, and there were no significant changes in responses to individual questions in either treatment group for sexual dysfunction over the course of the study. Neither the total scores nor the scores on individual questions showed an increase or decrease of sexual function in either group over time.

### 3.6 Perceived Stress Scale

Both groups reported moderate levels of perceived stress throughout the duration of the study. Mean baseline scores in the active and placebo groups were 20.8 and 21.3, respectively, both within the range of 14–26 defined as moderate stress per the PSS.

When comparing within groups, the active group showed a significant decrease from baseline to Day 180 (mean score = 19.3, *p* < 0.01), whereas the placebo group did not change significantly (mean score = 20.4, *p* > 0.05). When looking at individual questions, a greater proportion of subjects reported positive ratings in the active group compared to the placebo for all questions at Day 180, and three questions reached statistical significance for higher positive ratings in the active group compared to placebo (all *p* < 0.05). Overall, while the active group reported slightly improved perceived stress scores, both groups remained largely in the “moderately stressed” category over the course of the study.

### 3.7 Additional Questionnaires

The self‐assessment questionnaires on hair parameters showed some improvements throughout the study, but the differences compared to placebo were not statistically significant. More subjects in the active group (83%) reported treatment satisfaction than the placebo group (66%), although this difference did not reach statistical significance (*p* > 0.05). At the final visit, over 90% of subjects in both groups reported that it was easy to add the capsules to their daily routine and that it was easier to take the four capsules once a day than to apply a topical product once or twice a day.

### 3.8 Safety

A total of eight possibly or probably related AEs in six subjects (three active, three placebo) were recorded over the course of the study, all linked to gastrointestinal upset such as bloating, irritable stomach, diarrhea, and intermittent nausea, leading one subject from each group to withdraw. One subject was discontinued due to an unrelated AE, and all other AEs were resolved with no intervention. There were no serious AEs.

## 4 Discussion

This was a 6‐month multi‐center, randomized, double‐blind, placebo‐controlled study to evaluate the safety and efficacy of a supplement to improve hair growth and quality in men with thinning hair. The results of this study show statistically significant improvements in subjects taking the supplement compared to placebo in hair growth and quality when assessed through blinded investigator global assessment, as well as hair appearance and coverage in the MHGQ. Although the original primary end point data was not usable due to protocol deviations during the data collection, the improvements found in the Investigator Global Assessment and MHGQ in the active group compared to the placebo group in a blinded manner indicate an overall positive effect of the nutraceutical. In contrast, the results from the placebo group in the blinded investigator global assessment trended toward more subjects rated as “worsened” than the active group. In addition, subjects in the placebo group disagreed that the intervention slowed down their hair loss in the MHGQ. These two findings suggest that hair thinning progression over the course of the study in the placebo group was visible to both the dermatologists and subjects. Subject satisfaction with the study intervention was also higher in the active group compared to the placebo, and no changes in sexual dysfunction for either treatment group were documented.

Significant improvement in the active group compared to placebo in the blinded investigator global assessment supports the efficacy of the nutraceutical in promoting hair growth and quality. These results are consistent with studies that have evaluated similar formulas for women in different life stages, showing that targeting underlying root causes of hair thinning in populations through botanicals, vitamins, and minerals can improve hair growth and quality [6, 7, 8, 23]. And, while addressing male hair loss and thinning clinically is usually done through a combination of therapies, the study here provides support for the benefits in hair growth and quality of the nutraceutical alone [24]. The study population also included subjects with NH pattern Levels II, IIA, III, IIIv, and IV, and diverse races and ethnicities, indicating and supporting previous studies showing that the nutraceutical is effective in a diverse population of men with thinning hair [5].

The MHGQ, a validated questionnaire, also corroborated the blinded investigator global assessments indicating improvement in hair growth and appearance for subjects taking the supplement. For all questions, more subjects in the active group gave positive responses than the placebo group. Additionally, within the active group, more subjects responded positively rather than negatively to every question. This was not the case in the placebo group, in which there were more subjects who responded negatively than positively in three of seven questions. Statistically significant differences between the active group and placebo group were especially found in questions pertaining to maintaining their hair. These results support that changes over time in male hair thinning are progressive, and that addressing the underlying root causes may help prevent additional loss over time. These differences were not found in the subjective Hair Growth and Quality Assessment Questionnaire, suggesting the importance of a validated tool shown to accurately reflect changes in hair growth interventions over time in this population [19].

Recruitment for the study was started during the summer of 2021, with the final subject visits in April 2023, making the study period overlap with large and varying degrees of COVID‐19 infection levels [25]. Through standard AE reporting, subjects who were infected and tested for the COVID‐19 virus during the study were recorded (six in the active group and four in the placebo group). All cases were mild, and no subject was terminated from the study due to COVID‐19 illness, and investigators do not believe the current results were impacted. To assess the stressful impact of the pandemic, the PSS was used and did not show any significant trends or changes throughout the duration of the study [21].

Through the double‐blind design and placebo control group, this study supports the efficacy of the evaluated supplement for improving hair growth and quality in men with thinning hair. Future studies, including phototrichograms for metrics such as hair counts, would be useful to quantify additional hair growth parameters while taking the supplement.

In conclusion, the present study shows the beneficial results of a supplement formulated with botanicals, vitamins, and minerals for addressing hair thinning in men. It also supports that the supplement was safe and well tolerated. While genetics play a role in hair loss and thinning, other key root causes can be addressed with clinically studied nutraceutical ingredients. This formulation targeting six key root causes of hair thinning showed significant improvements in hair growth and quality compared to placebo.

## Author Contributions

N.B. and G.A. performed and supervised the assessments and data collection. P.K.F. aided in interpreting the results. A.H. contributed to the writing of the manuscript. I.R. contributed to the design and implementation of the study and aided in the writing and editing of the manuscript. All authors discussed the results and provided input for the manuscript.

## Ethics Statement

All investigations were performed in accordance with the rules of the Declaration of Helsinki of 1975, under the approval of an Institutional Review Board (Advarra, Columbia, MD, Pro00055025, July 1, 2021), and conducted in compliance with good clinical practice. All participants provided written, informed consent before engaging in any study‐related procedures.

## Consent

A photo release form was obtained from all subjects prior to their participation in the study.

## Conflicts of Interest

Dr. Neal Bhatia is an advisor for Nutrafol and has received research grants from Nutraceutical Wellness Inc. Dr. Glynis Ablon has previously received research grants from Nutraceutical Wellness Inc. Dr. Patricia K. Farris is an advisor for Nutrafol and Nutraceutical Wellness Inc. Drs. Adina Hazan and Isabelle Raymond are employees of Nutraceutical Wellness Inc.

## Data Availability

The data that support the findings of this study are available from the corresponding author upon reasonable request.
